# Concurrent sexual partnerships and associated factors: a cross-sectional population-based survey in a rural community in Africa with a generalised HIV epidemic

**DOI:** 10.1186/1471-2458-11-651

**Published:** 2011-08-17

**Authors:** Dermot Maher, Laban Waswa, Alex Karabarinde, Kathy Baisley

**Affiliations:** 1 Medical Research Council/Uganda Virus Research Institute (MRC/UVRI) Uganda Research Unit on AIDS, Entebbe, Uganda; 2 Department of Epidemiology and Population Health, London School of Hygiene and Tropical Medicine, London, UK

## Abstract

**Background:**

Although concurrent sexual partnerships may play an important role in HIV transmission in Africa, the lack of an agreed definition of concurrency and of standard methodological approaches has hindered studies. In a long-standing general population cohort in rural Uganda we assessed the prevalence of concurrency and investigated its association with sociodemographic and behavioural factors and with HIV prevalence, using the new recommended standard definition and methodological approaches.

**Methods:**

As part of the 2010 annual cohort HIV serosurvey among adults, we used a structured questionnaire to collect information on sociodemographic and behavioural factors and to measure standard indicators of concurrency using the recommended method of obtaining sexual-partner histories. We used logistic regression to build a multivariable model of factors independently associated with concurrency.

**Results:**

Among those eligible, 3,291 (66%) males and 4,052 (72%) females participated in the survey. Among currently married participants, 11% of men and 25% of women reported being in a polygynous union. Among those with a sexual partner in the past year, the proportion reporting at least one concurrent partnership was 17% in males and 0.5% in females. Polygyny accounted for a third of concurrency in men and was not associated with increased HIV risk. Among men there was no evidence of an association between concurrency and HIV prevalence (but too few women reported concurrency to assess this after adjusting for confounding). Regarding sociodemographic factors associated with concurrency, females were significantly more likely to be younger, unmarried, and of lower socioeconomic status than males. Behavioural factors associated with concurrency were young age at first sex, increasing lifetime partners, and a casual partner in the past year (among men and women) and problem drinking (only men).

**Conclusions:**

Our findings based on the new standard definition and methodological approaches provide a baseline for measuring changes in concurrency and HIV incidence in future surveys, and a benchmark for other studies. As campaigns are now widely conducted against concurrency, such surveys and studies are important in evaluating their effectiveness in decreasing HIV transmission.

## Background

An effective response to the HIV epidemic is a global priority, and sub-Saharan Africa faces the greatest challenge as the region with the largest share of the global HIV burden and the fewest resources. In many countries in sub-Saharan Africa the HIV epidemic is generalized, and the extent to which concurrent sexual partnerships contribute to HIV transmission and explain differences in HIV prevalence between regions has implications for HIV prevention. The question as to the importance of concurrent sexual partnerships in driving the HIV epidemic in sub-Saharan Africa was initially raised in the early 1990s [1,2] and debate on the role of concurrency has continued since [3-5]. Much of our understanding about the effect of concurrent sexual partnerships on the spread of HIV derives from mathematical models, and evidence for the importance of concurrency in driving HIV transmission in the region is limited [6], and weakened by the variety of different measures for concurrency used in different studies [4]. Studies measuring the dynamics of concurrency and its association with HIV risk at the population and individual levels are urgently needed [7].

The expert consultation convened by UNAIDS in 2009 recommended standardized definitions and methodological approaches for measuring sexual concurrency, and the initiation of research studies to build the evidence base for an appropriate public health response [8]. Concurrency was defined as "overlapping sexual partnerships in which sex with one partner occurs between two episodes of sex with another partner"[8]. The recommended definition emphasises the occurrence of sustained overlapping partnerships rather than a single long-term partnership with the occasional one-off sexual encounter outside the partnership [8]. In addition to temporal overlap, an important consideration is the influence of the characteristics of the partners involved and of the type of sex (e.g. use of condoms) on risk of HIV transmission [8]. Polygamy represents a particular, institutionalized form of concurrency, which may in some ways deserve special consideration since polygamous marriages are likely to be less transient and more accurately reported than informal partnerships, and account for a substantial share of all concurrent partnerships in many countries in sub-Saharan Africa [9]. A large open general population cohort in southwest Uganda, which was established in 1989 for HIV surveillance [10,11] provided the opportunity to study concurrency. The aims were to assess the prevalence of concurrency and investigate its association with sociodemographic and behavioural factors and with HIV prevalence in a rural community with a generalised HIV epidemic, using the new internationally recommended definition and methodological approaches [12].

### Setting

Uganda has an estimated 30 million population, mostly engaged in subsistence agriculture. Annual Gross National Income is $340 per capita and mean life expectancy at birth is 51 years [13]. The study cohort comprises approximately 20,000 residents of 25 neighbouring villages in rural Uganda. About half of the residents are children aged 12 years and below. Most dwellings are distributed throughout the countryside rather than clustered in villages, which mainly represent administrative areas demarcated on maps rather than population centres. The study population are mostly subsistence farmers and levels of literacy are low [14]. There are no tarmac roads and access may be difficult during the rains. The community consists mostly of people from the Baganda tribe, with 15% being of Rwandese origin, who are well assimilated. Religious affiliation is mostly Christian, with a significant Muslim minority (28%). Marriage in Baganda society can take many forms, including customary, religious, civil and informal, and polygyny is common. Adult HIV prevalence in the cohort initially declined from 8.5% in 1991 to 6.2% in 2000, but then rose to 7.7% in 2005 [15].

## Methods

Full details of the cohort and annual HIV serosurveys have been published elsewhere [10,11]. In brief, an annual household survey has been conducted since 1989, with all study village residents aged ≥ 13 years eligible for inclusion. Community sensitization activities precede each survey. All households are visited by, in turn, the mapping, census and survey teams (all accompanied by a village councillor). Consenting residents are interviewed at home in the local language by trained survey staff using a standardised questionnaire (see additional files 1 and 2) to collect information on socio-demographic and behavioural characteristics, and provide a blood sample for HIV testing. The survey teams comprise men and women who interview participants without matching of participant to interviewer by age or sex, in a location in the compound chosen to assure confidentiality. Average annual survey participation is about 60%-65%, although an estimated 84% have ever participated. Two enzyme immunoassays are used with set algorithms to establish HIV serostatus [16]. All participants in the general population cohort are encouraged to learn their HIV status at local voluntary counselling and testing centres. Those who are found in the suvey to have HIV infection are offered referral to the study clinic [17], which introduced ART in 2004 [18].

**Figure 1:**
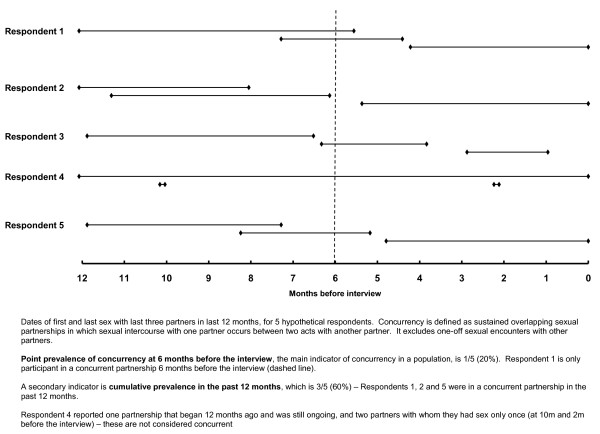
**Sexual partner histories of 5 hypothetical participants to illustrate how concurrency is defined in Survey Round 21**.

As part of the 2010 annual HIV serosurvey, we measured standard indicators of concurrency using the recommended method of survey of sexual-partner histories [12]. Detailed data on the last three sexual partnerships in the past 12 months were collected using a structured questionnaire (see additional files 1 and 2) to identify partnerships in which the dates of first and last sex overlapped. Participants were asked about the first and last time they had sex with each partner, the duration and type of relationship (e.g. spouse, casual, commercial), whether they used a condom at last sex, and if the relationship was still ongoing. Sexual partnerships lasting one day were not considered concurrent, even if they occurred within the period of another partnership (Figure 1) [8].

Questionnaire information also included sociodemographic and behavioural factors. Sociodemographic factors included age, sex and socioeconomic status (SES), which was measured using an asset index, created by combining data on 22 household possessions using principal component analysis. Behavioural characteristics included age at first sex, number of partners in the past year and lifetime, and casual partner in past year. Problem drinking was defined as an Alcohol Use Disorders Identification Test score above 8 in men or above 7 in women [19]. Information on HIV serostatus was obtained from the 2010 survey, or from the last available survey result if a participant consented to the questionnaire but declined an HIV test. For participants with a spouse resident in the study area, information on their spouse's HIV status was obtained through linkage using a unique spousal identification number.

### Data collection and analysis

Data were double-entered and verified in Access and analysed using Stata 10 (Stata Corporation, College Station, USA). We calculated three indicators of concurrency: 1) the proportion of participants in a concurrent partnership six months before the interview (point prevalence); 2) the proportion of participants in a concurrent partnership at any time during the past year ('cumulative prevalence'); and 3) the proportion of participants in a concurrent partnership involving a non-spousal partner in the past year (i.e. excluding polygynous partners).

We investigated sociodemographic and behavioural factors associated with cumulative prevalence of concurrency among those reporting a partner in the past year. Analyses were stratified by sex because it was thought *a priori *that some associations might differ between males and females. We used logistic regression to build a multivariable model of factors independently associated with concurrency. Potential determinants of concurrency were examined using a conceptual framework. Sociodemographic factors whose association with concurrency was significant at p < 0.10 were included in a multivariable model. Those remaining independently associated with concurrency at p < 0.10 were retained in a core model. Behaviour factors were added to this core model one by one. Those whose adjusted association with concurrency reached significance at p < 0.10 were included in a multivariable model; those remaining significant at p < 0.10 were retained. Lastly, factors were allowed to drop from the model until all remaining in the final model were associated at p < 0.10.

We examined the association of concurrency in the past year with HIV prevalence, among those reporting a partner in the past year. To assess whether concurrency was associated with an individual's HIV status after controlling for other characteristics of the partnership, we considered the following potential confounders: age, SES, marital status, age at first sex, lifetime partners, casual partner in past year and condom use at last sex. Variables that changed the crude odds ratio (OR) for the association of concurrency and HIV status by more than 10% were retained in an adjusted model. Lastly, we did a similar analysis among participants who had a current partner in the study area, with partner's HIV status as the outcome, using random effects logistic regression to account for correlation between spouses in polygynous unions.

### Ethical issues

The study was approved by the Science and Ethics Committee of the Uganda Virus Research Institute and by the Uganda National Council for Science and Technology.

## Results

### Characteristics of cohort participants

At census in 2010, there were 4,968 males and 5,598 females aged ≥ 13 years resident in the study area and eligible as survey participants. Of those, 3,291 (66%) males and 4,052 (72%) females responded to the survey questionnaire. Participation was lower among males and females aged under 20, and among females 60 years and older. The mean (SD) age of responders was 31 (18) years for males and 33 (17) years for females (Table 1). Non-responders were of higher SES than responders, with 26% of non-responders in the highest SES quintile compared with 23% of responders (p < 0.01). Non-responders were also more likely to be HIV-positive than responders (8% versus 6%, p < 0.01).

**Table 1:** Description of study participants in survey

	Males (N = 3,291)	Females (N = 4,052)
**SOCIO-DEMOGRAPHIC/ECONOMIC FACTORS**		
**Age (years)^1^**		
< 20	1278 (38.8%)	1222 (30.2%)
20-29	598 (18.2%)	830 (20.5%)
30-39	489 (14.9%)	721 (17.8%)
40-49	375 (11.4%)	526 (13.0%)
50-59	243 (7.4%)	343 (8.5%)
≥ 60	308 (9.4%)	410 (10.1%)
**Marital status**		
Never married	1678 (51.0%)	1366 (33.7%)
Married with 1 wife	1163 (35.3%)	1361 (33.6%)
Married with > 1 wife	148 (4.5%)	464 (11.5%)
Separated/divorced/widowed	302 (9.2%)	861 (21.2%)
**Age at first marriage (if ever married)^1^**		
< 16 years	9 (0.6%)	403 (15.0%)
16-19 years	292 (18.1%)	1534 (57.2%)
20-24 years	703 (43.6%)	539 (20.1%)
25+ years	552 (34.2%)	125 (4.7%)
Doesn't remember	56 (3.5%)	81 (3.0%)
Median (IQR) age at first marriage (years)	22 (20-25)	18 (16-20)
**Education level^1^**		
Less than primary	193 (5.9%)	463 (11.4%)
Incomplete primary	1565 (47.6%)	1677 (41.4%)
Primary	658 (20.0%)	823 (20.3%)
Junior/secondary	720 (21.9%)	935 (23.1%)
Above secondary	153 (4.6%)	153 (3.8%)
**SES score tertile^1^**		
Low	852 (27.8%)	953 (25.4%)
Middle	1085 (35.5%)	1396 (37.2%)
High	1123 (36.7%)	1407 (37.5%)

**SEXUAL BEHAVIOUR, AMONG THOSE WHO HAVE EVER HAD SEX**		
	**(N = 2100)**	**(N = 3024)**
**Age at first sex (years)^2^**		
< 15	163 (7.8%)	360 (11.9%)
15-16	395 (18.9%)	1028 (34.0%)
17-18	659 (31.4%)	1044 (34.5%)
19-20	405 (19.3%)	332 (11.0%)
21+	205 (9.8%)	67 (2.2%)
Doesn't remember	269 (12.8%)	192 (6.4%)
Median (IQR) age at first sex (years)	18 (16-20)	17 (15-18)
**Partners in past year^2^**		
None	431 (20.5%)	949 (31.4%)
1	1241 (59.1%)	2038 (67.4%)
2	300 (14.3%)	33 (1.1%)
3+	128 (6.1%)	3 (0.1%)
**Lifetime partners^2^**		
1	245 (11.7%)	985 (32.7%)
2	264 (12.7%)	940 (31.2%)
3-4	476 (22.8%)	842 (28.0%)
5+	928 (44.5%)	214 (7.1%)
Doesn't remember	174 (8.3%)	30 (1.0%)
Median (IQR) lifetime partners	4 (2-8)	2 (1-3)
**Last time had sex with any partner^2^**		
In last month	1388 (66.1%)	1642 (54.3%)
2-6 months	227 (10.8%)	342 (11.3%)
7-12 months	53 (2.5%)	90 (3.0%)
> 12 months	431 (20.5%)	949 (31.4%)
**Sex with an extramarital partner in past year^3^**		
Yes	201 (15.3%)	17 (0.9%)

**CONCURRENCY INDICATORS, AMONG THOSE HAVING SEX IN PAST YEAR**		
	**(N = 1668)**	**(N = 2074)**
**Concurrency at 6m before interview**		
Yes	280 (16.8%)	11 (0.5%)
**Any concurrency in last 12 m**		
Yes	340 (20.4%)	19 (0.9%)
**Any concurrency in last 12 m among those with > 1 partner^4^**		
Yes	340 (79.6%)	19 (52.8%)
**Any concurrency in last 12 m, excluding polygynous partnerships**		
Yes	230 (13.8%)	19 (0.9%)

**CONCURRENCY INDICATORS, AMONG ALL AGED 15-49**		
	**(N = 2273)**	**(N = 2855)**
**Concurrency at 6 m before interview**		
Yes	223 (9.8%)	11 (0.4%)
**Any concurrency in last 12 m**		
Yes	281 (12.4%)	19 (0.7%)
**Any concurrency in last 12 m, excluding polygynous partnerships**		
Yes	211 (9.3%)	19 (0.7%)
**HIV STATUS, AMONG ALL PARTICIPANTS^5^**		
Negative	3124 (95.5%)	3731 (92.6%)
Positive	148 (4.5%)	298 (7.4%)

^1^Missing data on age married for 1 male and 4 females. Missing data on education for 2 males and 1 female. Missing SES index for 231 males and 296 females

^2^Missing data on age at first sex for 4 males and 1 female. Missing data on lifetime partners for 13 males and 13 females. Missing data on last sex for 1 male and 1 female.

^3^Among 1311 males and 1825 females who are currently married.

^4^Among 427 males and 36 females reporting > 1 partner in past 12 months.

^5^Missing HIV status for 19 males and 23 females.

Over a third of survey participants were currently married (40% of men and 45% of women), and among those, 11% of men and 25% of women reported being in a polygynous union. No women reported being in a union involving more than one husband. Median age at first marriage was 22 years for men and 18 years for women. Most participants had ever been sexually active (64% of men and 75% of women), with a median age at first sex of 18 years (men) and 17 years (women). Among participants who had ever been sexually active, 1,669 (79%) men and 2,074 (69%) women reported at least one partner in the past 12 months. Over half (54%) of the men, but only 15% of women reported having had 4 or more lifetime partners. HIV seroprevalence was 4.5% in men and 7.4% in women.

Among married participants, 201 (15%) men and 17 (0.9%) women reported having sex with an extramarital partner in the past 12 months. Men in polygynous unions were less likely to report an extramarital partner in the past year than those in unions with one wife (7.4% versus 16.3% respectively, p = 0.005). The proportion of married women who reported extramarital partnerships was similar for those in polygynous and those in monogynous marriages (1.1% versus 0.9% respectively, p = 0.70).

### Prevalence and characteristics of concurrency

We present results for overall concurrency, for polygynous concurrency and for concurrency excluding polygyny (non-spousal concurrency).

#### Overall concurrency

Among those reporting a sexual partner in the past year, 280 (17%) men and 11 (0.5%) women were in a concurrent partnership six months before the interview (Table 1). The cumulative prevalence of concurrency during the past year was 20% (N = 340) in men and 0.9% (N = 19) in women. Among participants who reported more than one partner in the past year, the cumulative prevalence of concurrency was 80% in men and 53% of women. Among all men, 32% of concurrency was accounted for by polygyny. The majority of concurrent relationships involved spouses or regular partners only (81% of men and 74% of women; Table 2).

**Table 2:** Characteristics of those with any concurrency among those reporting a sexual partner in past 12 months

	Males (N = 340)	Females (N = 19)
**Number of overlapping partnerships**		
Two	283 (83.2%)	19 (100%)
Three	57 (16.8%)	0
**Type of relationships^1^**		
All are spouses (polygynous)	110 (32.4%)	-
Spouse and/or regular partners only	171 (50.3%)	14 (73.7%)
Spouse/regular with casual partner	53 (15.6%)	4 (21.1%)
All are casual partners	6 (1.8%)	0
**Concurrent partnership still ongoing**		
Yes	247 (72.7%)	8 (42.1%)
**Condom use at last sex with concurrent partners**		
Did not use with any	206 (60.6%)	11 (57.9%)
Used with some	108 (31.8%)	5 (26.3%)
Used with all	26 (7.6%)	3 (15.8%)
**Condom use at last sex by type of relationship**		
**Among those with spousal concurrent partners only**		
Did not use with any	100 (90.9%)	-
Used with some	9 (8.2%)	-
Used with all	1 (0.9%)	-
**Among those with regular non-spousal concurrent partners^2^**		
Did not use with any	92 (53.8%)	7 (50.0%)
Used with some	64 (37.4%)	4 (28.6%)
Used with all	15 (8.8%)	3 (21.4%)
**Among those with a casual concurrent partner^3^**		
Did not use with any	14 (23.7%)	3 (75.0%)
Used with some	35 (59.3%)	1 (25.0%)
Used with all	10 (16.9%)	0
**HIV seropositive^4^**		
Overall	28 (8.2%)	7 (36.8%)
Among those with 2 concurrent partnerships	23 (8.2%)	7 (36.8%)
Among those with 3 concurrent partnerships	5 (8.8%)	-
Among those with spousal concurrent partners only	4 (3.7%)	-
Among those with regular non-spousal concurrent partners	16 (9.4%)	5 (35.7%)
Among those with a casual concurrent partner	8 (13.8%)	2 (50.0%)
**HIV seroprevalence in spouse^5^**		
Overall	25 (8.3%)	4 (66.7%)
Among those with 2 concurrent partnerships	21 (8.5%)	4 (66.7%)
Among those with 3 concurrent partnerships	4 (7.4%)	-
Among those with spousal concurrent partners only	15 (9.0%)	-
Among those with regular non-spousal concurrent partners	8 (7.4%)	3 (60.0%)
Among those with a casual concurrent partner	2 (7.7%)	1 (100%)

^1^Missing information on type of partner for 1 woman.

^2^Among those with any regular non-spousal concurrent partners but no casual partners (N = 171).

^3^Among those with any casual concurrent partners (N = 59).

^4^Missing HIV status for 2 men.

^5^Among those with a spouse in the study population: 301 wives of 230 men in concurrent partnerships, and 6 husbands of 6 women in concurrent partnerships.

Most individuals reported not using a condom at last sex with any of their concurrent partners (61% of men and 58% of women). Condom use was highest when concurrency involved a casual partner, with 17% of men reporting using a condom at last sex with all partners. HIV prevalence was 8% (28/340) in men and 37% (7/19) in women reporting any concurrent partnership in the past 12 months, compared with 6% of men and 9% of women with no concurrency (p = 0.22 and p < 0.001, respectively).

#### Polygynous concurrency

Among 148 men in polygynous marriages, 110 (74%) reported concurrency within their marriage only. Reported condom use was very low, with 91% of men reporting not using a condom at last sex with any partner. HIV prevalence was lower among men whose partnerships involved only polygyny than among those in non-spousal concurrent partnerships (4% vs 10%, p = 0.03).

#### Concurrency excluding polygyny

When polygynous concurrency was excluded, 171 (10%) men reporting sex in the past year were in concurrent partnership six months before the interview. The cumulative prevalence of non-spousal concurrency was 14% (N = 230). The majority of concurrent partnerships (74%) involved regular partners only. Slightly less than half (46%) reported not using a condom at last sex with any of their concurrent partners. HIV prevalence varied by partner type, and was highest among those with casual concurrent partners (14% vs 9% among those with regular concurrent partners, p = 0.34).

### Factors associated with concurrency

#### Overall concurrency

Because the prevalence of concurrency was so low in women (19 women, < 1%), the analysis of factors associated with concurrency was restricted to men. Sociodemographic factors associated with concurrency were older age, young age at first marriage, higher education and higher SES (Table 3). Behavioural factors associated with concurrency were young age at first sex, increasing lifetime partners, having a casual partner in the past year, and problem drinking. There was no evidence of an association of HIV prevalence with concurrency (p = 0.22), or of an association with spouse HIV-positivity (p = 0.51).

**Table 3:** Factors associated with concurrency in past 12 months, among 1668 men who report having had sex in the past 12 months

	Any concurrency		Concurrency excluding polygyny	
	
	n reporting/ N (%)	Unadjusted OR (95% CI)	n reporting/ N (%)	Unadjusted OR (95% CI)
**SOCIO-DEMOGRAPHIC/ECONOMIC FACTORS**				
**Age (years)**		P < 0.001		P < 0.001
< 30	92/537 (17.1%)	1	84/537 (15.6%)	1
30-39	100/440 (22.7%)	1.42 [1.04, 1.95]	81/440 (18.4%)	1.22 [0.87, 1.70]
40-49	89/326 (27.3%)	1.82 [1.30, 2.53]	46/326 (14.1%)	0.89 [0.60, 1.31]
50+	59/365 (16.2%)	0.93 [0.65, 1.33]	19/365 (5.2%)	0.30 [0.18, 0.50]
**Marital status**		P < 0.001		P = 0.08
Never married	47/295 (15.9%)	1.17 [0.82, 1.66]	47/295 (15.9%)	1.17 [0.82, 1.66]
Married with 1 wife	155/1108 (14.0%)	1	155/1108 (14.0%)	1
Married with > 1 wife	120/143 (83.9%)	32.1 [19.9, 51.7]	10/143 (7.0%)	0.46 [0.24, 0.90]
Separated/divorced/widowed	18/122 (14.8%)	1.06 [0.63, 1.80]	18/122 (14.8%)	1.06 [0.63, 1.80]
**Age at first marriage^1^**		P < 0.01		P = 0.11
22+ years	146/774 (18.9%)	1	94/774 (12.1%)	1
< 22 years	141/559 (25.2%)	1.45 [1.12, 1.89]	85/559 (15.2%)	1.30 [0.95, 1.78]
**Education level**		P = 0.04		P = 0.20
Incomplete primary or less	125/709 (17.6%)	1	87/709 (12.3%)	1
Primary	103/436 (23.6%)	1.45 [1.08, 1.94]	70/436 (16.1%)	1.37 [0.97, 1.92]
Junior or above	112/522 (21.5%)	1.28 [0.96, 1.70]	73/522 (14.0%)	1.16 [0.83, 1.62]
**SES score tertile**		P < 0.001		P = 0.002
Low	51/384 (13.3%)	1	32/384 (8.3%)	1
Middle	121/571 (21.2%)	1.76 [1.23, 2.51]	86/571 (15.1%)	1.95 [1.27, 2.99]
High	129/553 (23.3%)	1.99 [1.39, 2.83]	83/553 (15.0%)	1.94 [1.26, 2.99]

**SEXUAL BEHAVIOUR**				
**Age at first sex (years)^1^**		P < 0.001		P < 0.001
18+ years	134/802 (16.7%)	1	89/802 (11.1%)	1
< 18 years	160/645 (24.8%)	1.64 [1.27, 2.13]	117/645 (18.1%)	1.78 [1.32, 2.39]
**Lifetime partners^1^**		P < 0.001		P < 0.001
< 5 males	64/721 (8.9%)	1	43/721 (6.0%)	1
5+ males	235/793 (29.6%)	4.32 [3.21, 5.83]	163/793 (20.6%)	4.08 [2.87, 5.81]
Can't remember	40/147 (27.2%)	3.84 [2.46, 5.99]	23/147 (15.6%)	2.92 [1.70, 5.03]
**Condom use with last partner**		P = 0.82		P = 0.01
No	296/1444 (20.5%)	1	187/1444 (12.9%)	1
Yes	43/217 (19.8%)	0.96 [0.67, 1.37]	42/217 (19.0%)	1.61 [1.11, 2.34]
**Casual partner in past 12 m**		P < 0.001		P < 0.001
No	272/1480 (18.4%)	1	162/1480 (10.9%)	1
Yes	68/188 (36.2%)	2.52 [1.82, 3.48]	68/188 (36.2%)	4.61 [3.28, 6.47]
**Problem drinking**		P < 0.01		P = 0.001
No	312/1590 (19.6%)	1	208/1590 (13.1%)	1
Yes	26/74 (35.1%)	2.22 [1.36, 3.63]	21/74 (28.4%)	2.63 [1.56, 4.45]
**HIV serostatus**		P = 0.22		P = 0.02
Negative	310/1543 (20.1%)	1	205/1543 (13.3%)	1
Positive	28/112 (25.0%)	1.33 [0.85, 2.07]	24/112 (21.4%)	1.78 [1.11, 2.86]
**Spouse's HIV serostatus^2^**		P = 0.54		P = 0.98
Negative	276/1049 (26.3%)	1	124/1049 (11.8%)	1
Positive	25/85 (29.4%)	1.17 [0.72, 1.92]	10/85 (11.8%)	0.99 [0.50, 1.96]

^1^Cutpoints based on median values in males in the entire cohort (see Table 1)

^2^Among 1134 wives of 1056 men who had a spouse in the study population

Independent sociodemographic factors associated with concurrency were age (highest among those 30-39 years, and lowest in those aged over 50), being in a polygynous union, and higher SES (Table 4). Behavioural factors that were independently associated with concurrency were increasing lifetime partners (adjusted OR = 4.30, 95% CI = 2.95-6.27, comparing those above the cohort median lifetime partners versus those at median or below), and having a casual partner in the past 12 months (adjusted OR = 3.04, 95% CI = 1.94-4.75).

**Table 4:** Sociodemographic and behavioural factors associated with any concurrency in past 12 months, among 1,668 men who report having had sex in the past 12 months

	Any concurrency	Concurrency excluding polygyny
	
	Adjusted OR^1^ (95% CI)	Adjusted OR^1^ (95% CI)
**SOCIO-DEMOGRAPHIC/ECONOMIC FACTORS**		
**Age (years)**	**P < 0.001**	**P < 0.001**
< 30	**1**	**1**
30-39	**1.29 [0.81, 2.05]**	**1.21 [0.77, 1.93]**
40-49	**0.97 [0.57, 1.62]**	**0.85 [0.50, 1.43]**
50+	**0.39 [0.22, 0.70]**	**0.27 [0.14, 0.51]**
**Marital status**	**P < 0.001**	**P = 0.07**
Never married	**0.88 [0.51, 1.51]**	**0.79 [0.46, 1.36]**
Married with 1 wife	**1**	**1**
Married with > 1 wife	**43.5 [25.2, 74.8]**	**0.41 [0.19, 0.88]**
Separated/divorced/widowed	**0.76 [0.41, 1.40]**	**0.75 [0.40, 1.40]**
**Age at first marriage^1^**	P = 0.27	P = 0.29
22+ years	1	1
< 22 years	1.22 [0.86, 1.73]	1.22 [0.85, 1.77]
**Education level**	P = 0.61	P = 0.82
Incomplete primary or less	1	1
Primary	1.19 [0.81, 1.75]	1.07 [0.72, 1.60]
Junior or above	1.16 [0.79, 1.71]	1.14 [0.76, 1.69]
**SES score tertile**	**P = 0.01**	**P = 0.01**
Low	**1**	**1**
Middle	**1.72 [1.11, 2.65]**	**1.85 [1.17, 2.94]**
High	**1.85 [1.20, 2.87]**	**1.92 [1.21, 3.05]**

**SEXUAL BEHAVIOUR**		
**Age at first sex^2^**	P = 0.41	P = 0.41
18 years or older	1	1
< 18 years	1.16 [0.82, 1.63]	1.16 [0.81, 1.66]
**Lifetime partners^2^**	**P < 0.001**	**P < 0.001**
Less than 5	**1**	**1**
5 or more	**4.30 [2.95, 6.27]**	**4.55 [3.03, 6.82]**
Can't remember	**3.35 [1.82, 6.17]**	**4.05 [2.13, 7.69]**
**Condom use with last partner**	P = 0.14	P = 0.11
No	1	1
Yes	1.45 [0.89, 2.36]	1.51 [0.92, 2.47]
**Casual partner in past 12 months**	**P < 0.001**	**P < 0.001**
No	**1**	**1**
Yes	**3.73 [2.40, 5.82]**	**3.90 [2.13, 7.69]**
**Problem drinking**	P = 0.15	P = 0.14
No	1	1
Yes	1.61 [0.86, 3.00]	1.64 [0.87, 3.12]

^1^Adjusted for age, marital status, SES, lifetime partners and casual partner in past 12 m

^2^Cutpoints based on median values in males in the entire cohort (see Table 1)

#### Polygynous concurrency

Sociodemographic factors associated with polygynous concurrency were older age, young age at first marriage and higher SES (data not shown). Behavioural factors associated with polygynous concurrency were increasing lifetime partners and not using a condom at last sex. There was no evidence of an association of polygynous concurrency with HIV prevalence (p = 0.18), or with spouse HIV-positivity (p = 0.41).

Independent sociodemographic factors associated with polygynous concurrency were increasing age, higher education, early age at first marriage, and not using condoms at last sex (adjusted OR = 8.76, 95% CI = 1.20-64.1).

#### Concurrency excluding polygyny

Sociodemographic factors associated with non-spousal concurrency in the unadjusted analysis were age, not being in a polygynous union and high SES (Table 3). Behavioural factors associated with concurrency were young age at first sex, increasing lifetime partners, condom use with last partner, having a casual partner and problem drinking. Non-spousal concurrency was associated with HIV prevalence (p = 0.02), but not with spouse HIV-positivity (p = 0.99). In the multivariable analysis, the same factors were found to be independently associated with non-spousal concurrency as with overall concurrency, and the direction of effect was similar (Table 4), except for the association with marital status: men in polygynous unions were least likely to report non-spousal concurrency (7% versus 14% of men with only one wife; adjusted OR = 0.41, 95% CI = 0.19-0.88).

### Association of concurrency and HIV prevalence

#### Overall concurrency

We examined the association of concurrency with HIV prevalence only in men, since so few women reported concurrent partnerships. Although HIV prevalence was somewhat higher among men in concurrent partnerships than among those with no concurrency (8% versus 6%), there was no evidence of a strong association (unadjusted OR = 1.33, 95% CI = 0.85-2.08; Table 5). After adjusting for confounders, the effect of concurrency was completely attenuated (adjusted OR = 1.04, 95% CI = 0.62-1.77).

**Table 5:** Association of concurrency in past 12 months with HIV seroprevalence, unadjusted and after adjusting for confounders^1^

	Odds ratio (95% CI)	p-value
**OUTCOME = HIV SEROPREVALENCE** **EXPOSURE = ANY CONCURRENCY IN PAST 12 MONTHS**		
**Among men reporting sex in past 12 months (N = 1655)**		
**Model 1**		
Any concurrency in the past 12 months	1.33 (0.85-2.08)	P = 0.22
**Model 2**		
Any concurrency in the past 12 months	1.13 (0.67-1.89)	P = 0.64
Adjusted for: age group, marital status and lifetime partners^2^		
**Model 3**		
Any concurrency in the past 12 months	1.04 (0.62-1.77)	P = 0.88
Adjusted for: age group, marital status, lifetime partners^2 ^and casual partner in past 12 months		

**OUTCOME = HIV SEROPREVALENCE** **EXPOSURE = CONCURRENCY WITH POLYGYNOUS PARTNERS ONLY**		
**Among men reporting sex in past 12 months (N = 1655)**		
**Model 1**		
Concurrency with polygynous partner in past 12 months	0.50 (0.18-1.39)	P = 0.14
**Model 2**		
Concurrency with polygynous partner in past 12 months	0.40 (0.14-1.11)	P = 0.07
Adjusted for lifetime partners^2 ^in past 12 months		

**OUTCOME = HIV SEROPREVALENCE** **EXPOSURE = CONCURRENCY EXCLUDING POLYGYNY**		
**Among men reporting sex in past 12 months (N = 1655)**		
**Model 1**		
Concurrency with non-spousal partner in past 12 months	1.78 (1.11-2.86)	P = 0.02
**Model 2**		
Concurrency with non-spousal partner in past 12 months	1.36 (0.82-2.25)	P = 0.25
Adjusted for lifetime partners^2 ^and casual partner in past 12 months		

**OUTCOME = HIV SEROPREVALENCE IN SPOUSE** **EXPOSURE = ANY CONCURRENCY IN PAST 12 MONTHS**		
**Among wives of men reporting sex in past 12 months (N = 1134)**		
**Model 1**		
Any concurrency by husband in the past 12 months	1.17 (0.71-1.92)	0.54
**Model 2^3^**		
Any concurrency by husband in the past 12 months	0.97 (0.59-1.59)	0.90
Adjusted for husband's lifetime partners^2 ^		

^1^The following potential confounders were considered: age, SES, marital status, age at first sex, lifetime partners, casual partner in past year and condom use at last sex. Those variables which changed the OR for the association with concurrency by > 10% were retained.

^2^Lifetime partners above the median for males in the cohort (see Table 1)

^3^Adjusted model considered all potential confounders listed in footnote 1, and reported behaviour of wife (lifetime partners, casual partner in past year, and condom use at last sex).

There was no evidence of an association between overall concurrency and HIV prevalence among spouses, either in the unadjusted or adjusted analysis (adjusted OR = 0.97, 95% CI = 0.59-1.60, p = 0.44).

#### Polygynous concurrency

HIV prevalence was lower among men in polygynous concurrent partnerships only than among men reporting no or non-spousal concurrency (4% vs 7%), although that association was not statistically significant (Table 5). After adjusting for confounders, there was some evidence that men in polygynous partnerships were less likely to be HIV positive (adjusted OR = 0.40, 95% CI = 0.14-1.11; p = 0.07). There was no evidence of an association between polygynous concurrency and HIV prevalence among spouses, either in the unadjusted or adjusted analysis (adjusted OR = 1.11, CI = 0.61-2.03, p = 0.72)

#### Concurrency excluding polygyny

When concurrency involving polygynous partners was excluded, there was some evidence of an association between concurrency and HIV prevalence (unadjusted OR = 1.78, 95% CI = 1.11-2.86; p = 0.02). However, after adjusting for confounders, this association was greatly attenuated (adjusted OR = 1.36, 95% CI = 0.82-2.25, p = 0.25). There was no evidence of an association between non-spousal concurrency and HIV prevalence among spouses, either in the unadjusted or adjusted analysis (adjusted OR = 0.83, 95% CI = 0.41-1.66, p = 0.59).

## Discussion

The prevalence of overall concurrency was much higher in males than in females, for all of the indicators that we assessed. Polygyny accounted for a third of concurrency in men. Among men there was no evidence of an association between overall concurrency and HIV prevalence. However, HIV prevalence varied by type of concurrent partnership, and was higher among men whose concurrent partnerships involved a casual partner than in those whose concurrent partnerships only involved spouses and regular partners. Among women, although HIV prevalence was strongly associated with concurrency, the number reporting concurrency was too low to explore this further.

Concurrency is generally thought to be associated not with an increased risk of HIV infection in an individual who has concurrent partners, but with increased risk of HIV transmission to that person's partners [20]. In our study we found that HIV prevalence was somewhat higher among men in concurrent partnerships than among those with no concurrency (8% versus 6%), but after adjusting for confounders the effect was completely attenuated. When polygyny was excluded, HIV prevalence was higher among men in concurrent partnerships than among those with no concurrency (10% versus 4%), although after adjusting for confounders that association was greatly attenuated and not statistically significant. We found no evidence of an association between men reporting any concurrency and higher HIV prevalence among their wives. However our cross-sectional survey does not allow assessment of the timing of concurrent partnerships in relation to the occurrence of HIV infection, which limits the value of our data in assessing individual HIV risk and HIV transmission.

As found elsewhere in many countries in sub-Saharan Africa, a substantial proportion of concurrent partnerships are polygynous marriages, and men whose concurrency is confined to polygyny may be at lower risk of HIV than men with no or non-spousal concurrency [9]. Although other studies of the relationship between polygyny and extramarital sex have been inconclusive [21,22], in our study polygynous men were significantly less likely than monogamous men to report extramarital partnerships or non-spousal concurrency. In addition to our finding of an association between HIV and partner type, we found that reported behaviours such as condom use varied by type of concurrent partnership. The finding that reported condom use among men was lowest when concurrency involved polygynous wives only, is consistent with the low levels of condom use with spouses usually reported in many settings.

The much higher prevalence of overall concurrency in males than in females is consistent with findings elsewhere. A systematic review of concurrency rates reported in Demographic and Health Surveillance (DHS) surveys between 1995 and 2009 in countries in sub-Saharan Africa and in other regions found a wide range between 1-16% in males and 0.1-3.2% in females [5]. A national survey in Botswana reported in 2010 a higher prevalence of concurrency among men and women (19% and 6% respectively) [23]. However, the gender difference in our study (20% of men and 0.9% of women reporting at least one concurrent partnership in the past year) is greater than has been reported elsewhere. This discrepancy is likely in part to reflect under-reporting of concurrent relationships by women. In population-based surveys on sexual behavior in a number of countries in Africa, e.g. Tanzania [24] and Zimbabwe [25], men consistently reported higher numbers of sexual partners than women, which may be associated with male exaggeration or female under-reporting.

Our estimated overall point prevalences of concurrency in males (10%) and females (0.4%) are similar to those reported in the neighbouring district of Rakai in 1994 (14% and 1.3% respectively) [26], although comparability is limited by the likelihood of considerable changes in sexual behaviour since 1994. Comparisons of the level and role of concurrency in HIV transmission in different populations are difficult. A variety of different measures for concurrency have been used in different studies [4], some of which do not even capture whether or not partnerships overlap [6], e.g. the proposed use of multiple partnerships in a short time period as a proxy measurement of concurrency [27]. The variety of definitions and indicators of concurrency is in part a reflection of the complexity of sexual behaviours underpinning concurrency [28].

Our study had a number of strengths. Firstly, so far as we are aware, it is the first population-based survey in sub-Saharan Africa to measure the prevalence of overall concurrency using the new internationally recommended definition of concurrency and methodological approaches [12]. Secondly, we examined polygyny as a separate and important form of concurrency. Thirdly, for participants currently married to a person resident in the study area, we were able to obtain linked information on their spouse's HIV status using a unique spousal identification number. Although it is important in studies of concurrency to assess a correlation between index case concurrency and their partner's HIV status, few studies have been able to do this because of the difficulty of enrolling both partners in a sexual relationship [29].

One limitation of our study is that the number of women reporting concurrency is too small to enable us to satisfactorily explore associations. Furthermore, the prevalence of concurrency among women (0.9% in the past year) may reflect under-reporting and therefore may not be representative of all women. Among both men and women, a general limitation of sexual behaviour surveillance is that it is prone to selection, recall, denial and social desirability biases [8]. In using the method of survey of sexual-partner histories recommended by UNAIDS [12], the measures used to minimise these problems included asking participants about the first and last time they had sex with each partner in terms of how long ago these events were and prompting the respondent with key events that occurred in the year prior to the interview [8]. Another measure which may help mitigate these problems is matching participants to interviewers by age and by gender.

There were two potential sources of bias arising from differences between those who responded to the survey questionnaire and those who did not. Firstly, HIV-positive people were less likely to respond than HIV-negative people, which may have led to an underestimation of the prevalence of concurrency or other factors associated with HIV. Secondly, non-responders were of slightly higher SES than responders, and since concurrency among men was more prevalent among those of high SES this may have led to slight underestimation of concurrency prevalence. Maintaining a high participation rate is a challenge in a long established community-based annual HIV serosurvey [15]. Some participants may decline participation because they have previously had a blood test for HIV, know their result and decide against a repeat test. Finally, we collected information in our survey on some of the recommended covariates which are associated with risk of HIV transmission, such as type of relationship and condom usage within the partnership [8], but not on coital frequency.

The use of standard definitions and measures of concurrency is important so that the contribution of concurrency to HIV transmission can be estimated and intervention approaches and outcomes can be reported and compared across settings [8], including through improved DHS surveys. The HIV prevention efforts in many countries in sub-Saharan Africa now include campaigns aimed at discouraging concurrent partnerships ("get off the sexual network"). The success of these campaigns in decreasing HIV transmission depends on firstly their effectiveness in changing this sexual behaviour in the context of the deep-rooted social, economic and cultural determinants of gender dynamics [30] and secondly the extent to which concurrency contributes to HIV transmission, both of which should be measured. A systematic review of the effectiveness of mass communication programmes to change sexual risk behaviour in developing countries found mixed results-a few studies yielded small to moderate effects, but others achieved no change [31]. Longitudinal population-based cohort studies can make an important contribution to assessment of the importance of concurrency in HIV transmission. They allow a more detailed sexual partner history than in DHS and enable the measurement of HIV incidence, thus allowing determination of the impact of concurrency on population-level HIV risk. A network of longitudinal population-based cohort studies in sub-Saharan Africa could meet the need for sufficiently large numbers of study participants to provide more definitive assessment of the role of concurrency by pooling data [32]. Our findings from the 2010 HIV survey provide a baseline from which to measure changes in concurrency and HIV incidence in future surveys which allow linking of respondents over time, and a benchmark against which the findings of other studies also using the standard definition and methodological approaches can be compared. Surveys of HIV incidence could enable measurement of the potential impact of changing levels of concurrency on HIV transmission.

## Conclusion

The role of concurrency as a driver of the HIV epidemic in sub-Saharan Africa is contentious. Our findings on the prevalence of concurrency and the contribution of polygyny are based on the new standard UNAIDS definition of concurrency and methodological approaches for measuring concurrency. Although our cross-sectional survey does not allow assessment of the timing of concurrent relationships in relation to HIV infection, measurement of changes in concurrency and HIV incidence in future surveys could help clarify the role of concurrency in HIV transmission.

## Competing interests

The authors declare that they have no competing interests.

## Authors' contributions

DM had the idea for the study, was responsible for study design and drafted the manuscript. AK was responsible for data collection, and LW and KB were responsible for data analysis. DM and KB interpreted the data. All authors contributed to successive revisions of the manuscript, and read and approved the final manuscript.

## Authors' Information

Dermot Maher, Senior Clinical Epidemiologist, MRC/UVRI Uganda Research Unit on AIDS.

Laban Waswa, Senior Data Manager, MRC/UVRI Uganda Research Unit on AIDS.

Alex Karabarinde, Survey Coordinator, MRC/UVRI Uganda Research Unit on AIDS.

Kathy Baisley, Statistician, London School of Hygiene and Tropical Medicine, London, UK.

## Pre-publication history

The pre-publication history for this paper can be accessed here:

http://www.biomedcentral.com/1471-2458/11/651/prepub

## Supplementary Material

Additional file 1 **Survey round 21 questionnaire for males**. The questionnaire used by field staff for collecting information from adult male study participants. Click here for file

Additional file 2 **Survey round 21 questionnaire for females**. The questionnaire used by field staff for collecting information from adult female study participants. Click here for file
