# Supplementary material for: Concurrent sexual partnerships and associated factors: a cross-sectional population-based survey in a rural community in Africa with a generalised HIV epidemic
Source: BMC Public Health. 2011 Aug 17;11:651. doi: 10.1186/1471-2458-11-651 (PMC3176218; doi:10.1186/1471-2458-11-651)
Supplement: Additional file 1 — Survey round 21 questionnaire for males. The questionnaire used by field staff for collecting information from adult male study participants. [file 1471-2458-11-651-S1.DOC]

# MRC/UVRI SURVEY (version 14.12.09) ROUND 21

# QUESTIONNAIRE FOR ADULTS (age 13 years and above)

# MALE

# PERSONAL IDENTIFIER INFORMATION

# DDEXAM MDEXAM YDEXAM

Interviewer name & code no.………………..…|__|__| INTCODE Date of interview: |__|__| |__|__| |__|__|__|__|

dd mm yyyy

STICKER with participant’s personal identifier information

Residence code: |__|__| VNO |__|__|__| HNO |__|__| STM

PARTICIPANT NAME: .......................................................NAMEC |__|__|__|__|__|__|__|__| IDNO

SEX |__| DOB |__|__| |__|__| |__|__|__|__| 🡪 If year of birth unknown, ask or estimate age (years) |__|__| AGE

1 = M dd mm yy

Information for survey clerks and data manager

a. If person listed on Enumeration List, indicate any differences in age, name etc.

b. Revised name:………………………………………………………. RVNAME

Revised date of birth: |__|__| DDOB |__|__| MDOB |__|__|__| YDOB or |__|__| AGER

Remarks: ...........................…………………………………………………………………...

#### EDUCATION, OCCUPATION AND LIVELIHOOD

1. Osoma mu ssomero obudde bwonna? Are you in full-time education? |__| PSCH

1 = yes, 2 = no, 3 = don’t know

***If yes,***

2. Oli ku ddaala ki? What level of education? |__| FTED

1 = pre-primary school; 2 = primary school; 3 = secondary school; 4 = higher education (e.g. college, university)

5 = vocational college

***If no***,

3. Okolaki okusobola okwebezaawo/okufuna ensimbi? What is your source of livelihood?

(use code list L) |__|__| OCCUP1

|__|__| OCCUP2

|__|__| OCCUP3

|__|__| OCCUP4

|__|__| OCCUP5

**For all participants**

#### 4. Olina buyigirize bwenkana ki? |__|__| LEV

#### What level of education are you at (if still in education) or did you reach (if finished education)?

99 = nil; 18 = preprimary; 1-7 = P1-P7; 8-10 = J1-J3; 11-16 = S1- S6; 17 = college/university; 19 = vocational college

**MARITAL STATUS**

Ngenda okukubuuza ebikwata ku bufumbo, bw’oba olina/wali obaddeko ne gwotwala nga omukyala wo.

I’m going to ask you about your marital status. This means if you have ever regarded someone as your spouse.

5. Wali owasizaako? Wali obaddeko n’omuntu gw’otwala nga mukyalawo? |**__**|EVM

Have you ever been married, that is, have you ever had someone you called your wife?

1 = yes, 2 = no, 3 = don’t know

***If no, go to question 8***

***If yes,***

6. Walina emyaka emeka lwe wasookera ddala okuwasa? **|__|__|** AGEMG

How old were you when you first got married? State age (years)

7. Kino kikwata ku mukyala yenna gwolina kati oba gwe walina mu biseera ebyemabega. Tandika n’omuntu gwe wakasemba okubeera naye ng’odda emabega.

This refers to any wife you have now, or have ever had. List all wives, starting with the most recent union and going back in time.

| Name of spouse | Village number (survey clerks to add) | IDNO | Spouse from study area  1 = yes,  2 = no,  3 = don’t know | Year of union  (if not  known, enter  code 8888) | *Type of union | #Current status of union | If union  ended, state  year  (if not known,  enter code 8888) | Last sex with this spouse: how many months ago (up to 12 or more) |
| --- | --- | --- | --- | --- | --- | --- | --- | --- |
| WHS1 | **|__|__|**  VNO1 | IDNO1 | |**__**|  STAREA1 | |__|__|__|__|  YUN1 | |**__**|  TUN1 | |**__**|  STUN1 | |__|__|__|__|  ENDUN1 | **|__|__|**  SSXO1 |
| WHS2 | **|__|__|**  VNO2 | IDNO2 | |**__**|  STAREA2 | |__|__|__|__|  YUN2 | |**__**|  TUN2 | |**__**|  STUN2 | |__|__|__|__|  ENDUN2 | **|__|__|**  SSXO2 |
| WHS3 | **|__|__|**  VNO3 | IDNO3 | |**__**|  STAREA3 | |__|__|__|__|  YUN3 | |**__**|  TUN3 | |**__**|  STUN3 | |__|__|__|__|  ENDUN3 | **|__|__|**  SSXO3 |
| WHS4 | **|__|__|**  VNO4 | IDNO4 | |**__**|  STAREA4 | |__|__|__|__|  YUN4 | |**__**|  TUN4 | |**__**|  STUN4 | |__|__|__|__|  ENDUN4 | **|__|__|**  SSXO4 |
| WHS5 | **|__|__|**  VNO5 | IDNO5 | |**__**|  STAREA5 | |__|__|__|__|  YUN5 | |**__**|  TUN5 | |**__**|  STUN5 | |__|__|__|__|  ENDUN5 | |  |  | | --- | --- |   SSXO5 |

*Type of union 1 = informal, 2 = kwanjula, 3 = religious ceremony, 4 = civil ceremony with legal certificate, 5 = other

#Current status of union 1 = still married, 2 = separated or divorced, 3 = spouse died, 4 = other

**SEXUAL BEHAVIOUR**

**Interviewer**: please read to participant.

Kakati njagala okukubuuza obubuuzo obukwata kubulamu bwo obw'ekyama. Nkukakasa nti byonna bye tukungaanya mu kunoonyereza kuno bikuumibwa nga bya kyaama.

I will now ask you a few personal questions. Please be assured that all information collected in this study is treated confidentially.

8. Walina emyaka emeka lwe wasookera ddala okutabagana n’omuntu mubikolwa ebyekyama? **|__|__|** AGESX

How old were you when you first had sex? State age (years) 88 = can’t remember, 99 = never had sex

***If “Never had sex” go to question 48***

9. Wasembayo ddi okutabagana n’omuntu mu bikolwa ebyekyama?

When was the last time you had sex? State the number of days, weeks, months or years ago.

1-7 days |**__**| LSTSEXD more than 1 week, up to 4 weeks |**__**| LSTSEXW

more than 1 month, up to 12 months **|__|__|** LSTSEXM more than 1 year **|__|__|** LSTSEXY

***If more than 1 year ago, go to question 44***

**Sex partners in last 12 months**

Ngenda kukubuuza ebibuuzo ebikwata ku bulamu bwo obwekyama mu bbanga eryo’mwaka gumu oguyise.

I am now going to ask you about sexual relationships you have had in the past 12 months.

## 10. Wakatabagana n'abantu bameka mubikolwa eby'ekyama (mu bbanga ely-emyezi 12 egiyise? Nga otwaliddemu

mukyalawo, muganzi wo nabo ab'ekiseera obuseera, okugeza nga kumbaga, nyimbe, disco oba ku kinyumu kyonna.

How many people have you had sex with in the last 12 months? This includes all sexual partners, whether your current main partner, regular girlfriend or casual partners. Casual partners are such persons you had sex with only once or twice for example at a party, disco or other social gathering. (If none, code = 99) **|__|__|** SPYR

Ebibuuzo abiddako bikwata ku muntu gwewasembayo okwegatta naye mu bikolwa ebyekyama (“partner A”), n’eyaddako (“partner B”), n’eyamuddirira (“partner C”).

The next questions concern the last person you had sex with (“partner A”), the second-to-last (“partner B”), and the third-to-last (“partner C”).

**Last sexual partner (“partner A”)**

Era ebibuuzo bino bikwata ku muntu gwewasembayo okwegatta naye mubikolwa ebyekyama.

11. Wasembayo ddi okwegatta n’omuntu ono? Wayiseewo emyezi emeka? **|__|__|** LASTSXPA

When did you last have sex with this partner? How many months ago?

12. Wasooka ddi okwegatta n’omuntu ono? Wayiseewo emyezi emeka? **|__|__|** FRSTSXPA

When did you first have sex with this partner? How many months ago?

13. Mukyayagalana? Is the relationship still ongoing? 1 = yes, 2 = no, 3 = don’t know |**__**| ONGPA

14. Kumulundi ogwasembayo okwegatta n’omuntu ono mwakozesa akapiira? |**__**| CONDPA

The last time you had sex with this person, was a condom used?

1 = yes, 2 = no, 3 = don’t know

15. Mwakozesanga akapiira n’omuntu ono buli mulundi lwemwegattanga mubikolwa eby’ekyama mu myezi kumi n’ebiri egiyise? |**__**| EVCONDPA

Did you use a condom every time you had sex with this person in the last 12 months?

1 = yes, 2 = no, 3 = don’t know

16. Omuntu ono omutwala ng’ani? |**__**| TYPEPA

How would you best describe your relationship with this person?

1 = spouse 2 = ex-spouse, 3 = non-spousal regular partner, 4 = casual partner, 6 = commercial

5 = other (specify………………………………………………………….) TYPEPASP

17. Wa ebbanga lyewamala / lyomaze nga weegatta n’omuntu ono mu bikolwa eby’ekyama?

State the duration of your sexual relationship with this person. (If sex only once with this person, record 1 day)

1-7 days |**__**| DURDPA more than 1 week, up to 4 weeks |**__**| DURWPA

more than 1 month, up to 12 months **|__|__|** DURMPA more than1 year **|__|__|** DURYPA

18. Omuntu ono muto/yali muto okkusinga, mukulu okkusinga oba mwenkana mu myaka? |**__**| AGEPA

Is/was this person younger than, older than you, or about the same age?

1 = less than 5 years younger, 2 = 5-10 years younger, 3 = more than 10 years younger,

4 = less than 5 years older, 5 = 5-10 years older, 6 = more than 10 years older,

7 = same age, 8 = don’t know

19. Omuntu ono alina / yalina emyaka emeka? **|__|__|** AGECPA

What is /was the age of this person? State the age in years (88 = don’t know)

20. Kumulundi ogwasembayo okwegatta n’omuntu ono mubikolwa eby’ekyama waliwo omu kummwe eyali anywedde omwenge? |**__**| ALCPA

The last time you had sex with this person, did you or this person drink alcohol?

1 = yes, 2 = no, 3 = don’t know

***If yes,***

21. Waliwo omu kummwe eyali atamidde? |**__**| DRUNKPA

Were you or your partner drunk at the time?

1 = respondent only, 2 = partner only, 3 = both, 4 = neither, 5 = don’t know

**Second-to-last sexual partner (“partner B”)**

Ngenda kukubuuza ebibuuzo ebikwata ku muntu addirira gwewasembayo okwegatta naye mu bikolwa ebyekyama.

22. Wasembayo ddi okwegatta n’omuntu ono? **|__|__|** LASTSXPB

When did you last have sex with this partner? How many months ago?

23. Wasooka ddi okwegatta n’omuntu ono? Wayiseewo emyezi emeka? **|__|__|** FRSTSXPB

When did you first have sex with this partner? How many months ago?

24. Mukyayagalana? Is the relationship still ongoing? 1 = yes, 2 = no, 3 = don’t know |**__**| ONGPB

25. Ku mulundi ogwasembayo okwegatta n’omuntu ono mubikolwa ebyekyama mwakozesa akapiira?

The last time you had sex with this person, was a condom used? |**__**| CONDPB

1 = yes, 2 = no, 3 = don’t know

26. Mwakozesanga akapiira n’omuntu ono buli mulundi lwemwegattanga mu bikolwa eby’ekyama mu myezi kumi neebiri egiyise?

Did you use a condom every time you had sex with this person in the last 12 months? |**__**| EVCONDPB

1 = yes, 2 = no, 3 = don’t know

27. Omuntu ono omutwala ng’ani? |**__**| TYPEPB

How would you best describe your relationship with this person?

1 = spouse 2 = ex-spouse, 3 = non-spousal regular partner, 4 = casual partner, 6 = commercial

5 = other (specify………………………………………………………….) TYPEPBSP

28. Wa ebbanga lyewamala / lyomaze nga weegatta n’omuntu ono mu bikolwa eby’ekyama?

State the duration of your sexual relationship with this person. (If sex only once with this person, record 1 day)

1-7 days |**__**| DURDPB more than 1 week, up to 4 weeks |**__**| DURWPB

more than 1 month, up to 12 months **|__|__|** DURMPB more than1 year **|__|__|** DURYPB

29. Omuntu ono muto/yali muto okkusinga, mukulu okkusinga oba mwenkana mu myaka? |**__**| AGEPB

Is/was this person younger than, older than you, or about the same age?

1 = less than 5 years younger, 2 = 5-10 years younger, 3 = more than 10 years younger,

4 = less than 5 years older, 5 = 5-10 years older, 6 = more than 10 years older,

7 = same age, 8 = don’t know

30. Omuntu ono alina / yalina emyaka emeka? **|__|__|** AGECPB

What is / was the age of this person? State the age in years (88 = don’t know)

31. Kumulundi ogwasembayo okwegatta n’omuntu ono mubikolwa eby’ekyama waliwo omu kummwe eyali anywedde ku mwenge?

The last time you had sex with this person, did you or this person drink alcohol? |**__**| ALCPB

1 = yes, 2 = no, 3 = don’t know

***If yes,***

32. Waliwo omu kummwe eyali atamidde? |**__**| DRUNKPB

Were you or your partner drunk at the time?

1 = respondent only, 2 = partner only, 3 = both, 4 = neither, 5 = don’t know

**Third-to-last sexual partner (“partner C”)**

Ngenda kukubuuza ebibuuzo ebikwata ku muntu addirira ow’okubiri gwewasembayo okwegatta naye mu bikolwa ebyekyama.

33. Wasembayo ddi okwegatta n’omuntu ono? Wayiseewo emyezi emeka? **|__|__|** LASTSXPC

When did you last have sex with this partner? How many months ago?

34. Wasooka ddi okwegatta n’omuntu ono? **|__|__|** FRSTSXPC

When did you first have sex with this partner? How many months ago?

35. Mukyayagalana? Is the relationship still ongoing? 1 = yes, 2 = no, 3 = don’t know |**__**| ONGPC

Ngenda kukubuuzayo ebibuuzo ebirala.

I’m now going to ask some more detailed questions.

36.Ku mulundi ogwasembayo okwegatta n’omuntu ono mu bikolwa eby’ekyama mwakozesa akapiira?

The last time you had sex with this person, was a condom used? |**__**| CONDPC

1 = yes, 2 = no, 3 = don’t know

37. Mwakozesanga akapiira n’omuntu ono buli mulundi lwemwegattanga mu bikolwa ebyekyama?

Did you use a condom every time you had sex with this person in the last 12 months? |**__**| EVCONDPC

1 = yes, 2 = no, 3 = don’t know

38. Omuntu ono omutwala ng’ani? |**__**| TYPEPC

How would you best describe your relationship with this person?

1 = spouse 2 = ex-spouse, 3 = non-spousal regular partner, 4 = casual partner, 6 = commercial

5 = other (specify………………………………………………………….) TYPEPCSP

39. Wa ebbanga lyewamala / lyomaze nga weegatta n’omuntu ono mu bikolwa eby’ekyama?

State the duration of your sexual relationship with this person. (If sex only once with this person, record 1 day)

1-7 days |**__**| DURDPC more than 1 week, up to 4 weeks |**__**| DURWPC

more than 1 month, up to 12 months **|__|__|** DURMPC more than1 year **|__|__|** DURYPC

40. Omuntu ono muto/yali muto okkusinga, mukulu okkusinga oba mwenkana mu myaka? |**__**| AGEPC

Is/was this person younger than, older than you, or about the same age?

1 = less than 5 years younger, 2 = 5-10 years younger, 3 = more than 10 years younger,

4 = less than 5 years older, 5 = 5-10 years older, 6 = more than 10 years older,

7 = same age, 8 = don’t know

41. Omuntu ono alina / yalina emyaka emeka? **|__|__|** AGECPC

What is / was the age of this person? State the age in years (88 = don’t know)

42. Kumulundi ogwasembayo okwegatta n’omuntu ono mubikolwa |**__**| ALCPC

ebye’kyama waliwo omu kummwe eyali anywedde ku mwenge?

The last time you had sex with this person, did you or this person drink alcohol?

1 = yes, 2 = no, 3 = don’t know

***If yes,***

43. Waliwo omu kummwe eyali atamidde? |**__**| DRUNKPC

Were you or your partner drunk at the time?

1 = respondent only, 2 = partner only, 3 = both, 4 = neither, 5 = don’t know

**Summary of duration of last three sexual relationships in past 12 months (partners A, B and C)**

For partner A, partner B and partner C, put a cross in the calendar box for the last and first sex. Draw a line between the two crosses to show the duration of the sexual relationship.

| 12 or more | 11 | 10 | 9 | 8 | 7 | 6 | 5 | 4 | 3 | 2 | 1 | Partner A |
| --- | --- | --- | --- | --- | --- | --- | --- | --- | --- | --- | --- | --- |

| 12 or more | 11 | 10 | 9 | 8 | 7 | 6 | 5 | 4 | 3 | 2 | 1 | Partner B |
| --- | --- | --- | --- | --- | --- | --- | --- | --- | --- | --- | --- | --- |

| 12 or more | 11 | 10 | 9 | 8 | 7 | 6 | 5 | 4 | 3 | 2 | 1 | Partner C |
| --- | --- | --- | --- | --- | --- | --- | --- | --- | --- | --- | --- | --- |

**Current main partner**

44. Olina omuntu gwotwala nga owenkalakkalira? **|__|** CMP

Do you have a current main partner (i.e. spouse or most important regular)?

1 = yes, 2 = no, 8 = don’t know/not sure

***If no, go to question 47***

***If yes,***

45. Omuntu gwotwala nga owe nkalakkalira mwawufu ku bano betwoggedeko waggulu? **|__|** CP

Is your current main partner (i.e. spouse or most important regular) someone other than Partner A, B or C?

1 = yes, 2 = no, 3 = don’t know

***If yes,***

46. Ddi lwe wasembayo okutabagana n’omuntu wo owenkalakkalira? **|__|** PLSXCP

Ayinza okuba mukyalawo oba muganziwo.

How long ago did you last have sex with your current main partner (i.e. your spouse or most important regular, non-casual partner)? For polygamous men, ask about last wife he had sex with.

1 = less than a week, 2 = 1 week or more but less than a month, 3 = 1 month or more but less than a year,

4 = 1 year up to 5 years, 5 = 6 or more years, 6 = no spouse or regular, non-casual partner, 7 = unsure

**Total number of partners in lifetime**

47. Wakegatta nabantu bameka mu bikolwa ebyekyama mubulamu bwo bwonna? **|__|__|__|** SPEVER

In total, how many different people have you had sex with in your lifetime?

**Type of sex other than a man and a woman having sex**

48. Bulijjo bwetwogera ku mbeera yo kwegatta mu bikolwa ebyekyama tutegeeza wakati w’omussajja n’omukazi. Usually when we ask about “having sex” we mean a man and a woman having sex, with penetrative vaginal intercourse.

Naye omusajja ayinza okwegatta ne musajja munne - omanyi oba kino kikolebwa mu kitundu kino? **|__|** MSM

A man may also have sex with another man – do you know of this happening in this community?

1 = yes, 2 = no, 3 = don’t know

49. A woman may also have sex with another woman – do you know of this happening in this community?

Omukazi ayinza okwegatta ne mukazi munne - omanyi oba kino kikolebwa mu kitundu kino? **|__|** WSW

1 = yes, 2 = no, 3 = don’t know

**HEALTH** (questions about depression and alcohol consumption)

Interviewer: Please read this to the participant:

MRC has mainly been finding out about HIV. However it’s also important to know about some other conditions in this community. So I’m now going to ask about some other conditions.

Ekitongole kibadde kiri nnyo ku kunoonyereza ku mukenenya naye ng’ate kyamugaso okumanya ku ndwadde endala eziri mu bantu. Kati ngenda kukubuuza ebikwata ku ndwadde endala.

**Mental health** (Hopkins symptom checklist)

50. Olukalala luno wansi bw’ebubonero oba obuzibu abantu abamu bw’ebateera okubera nabwo. Lusomere oyo abuuzibwa n’obwegendereza osse obubonero mulunyiriri olulagga bw’abadde yeewulira mu mwezi oguwedde nga mwotwalidde n’olwaleero.

Listed below are symptoms or problems that people sometimes have. Please read each one of them carefully to the respondent and write in the appropriate column the score that best describes how he or she has been feeling **in the last one month including today**.

| **Qn. No.** | **Depression symptoms**  **Obubonero bw’bulwadde bw’kweraliikirira** | **Not at all**  **Nedda**  **1** | **A little**  **Katonotono**  **2** | **Quite a bit**  **Nnyo**  **3** | **Extremely**  **Nnyo nnyo**  **4** | **N/A**  **0** |
| --- | --- | --- | --- | --- | --- | --- |
|  | Okuwulira obunafu, no’buyongobevu mumubiri Feeling low in energy, slowed down |  |  |  |  |  |
|  | Okwenenya okuyitiridde  Blaming yourself for things |  |  |  |  |  |
|  | Okukaaba amangu  Crying easily |  |  |  |  |  |
|  | Obutayagala oba obutanyumirwa kwetaba munsonga z’obufumbo  Loss of sexual interest or pleasure |  |  |  |  |  |
|  | Obutayagala kulya  Poor appetite |  |  |  |  |  |
|  | Obutafuna tulo  Difficulty falling asleep or staying asleep |  |  |  |  |  |
|  | Okubulwa essuubi  Feeling hopeless about future |  |  |  |  |  |
|  | Okunakuwala  Feeling sad |  |  |  |  |  |
|  | Okufuna ekiwuubaalo  Feeling lonely |  |  |  |  |  |
| 10. | Okwagala okwetta  Thoughts of ending your life |  |  |  |  |  |
| 11. | Okuwuulira nga onyigiriziddwa  Feeling of being trapped or caught |  |  |  |  |  |
| 12. | Okweraliikirira buli kintu  Worry too much about things |  |  |  |  |  |
| 13. | Obutanyumirwa buli kintu  Feeling no interest in things |  |  |  |  |  |
| 14. | Okwekaka mu buli kintu  Feeling everything is an effort |  |  |  |  |  |
| 15. | Okuwuuliranga tolina mugaso  Feeling of worthlessness |  |  |  |  |  |

**Total score** = |__|__|__| DEPSCORE (If total score is 31or more, offer the participant counselling and referral)

**Alcohol** (Alcohol Use Disorders Identification Test “AUDIT”)

51. Soma ebibuuzo nga bwebiwandiikiddwa. Ebiddibwamu biwandiike n’obwegendereza. Tandika nga ogamba “kaakati ngenda kukubuuzaayo ebibuuzo ebimu ebikwatagana n’engeri gyobadde okozesamu ebyokunywa ebitamiiza mu mwaka oguyise.” Nnyonnyola eby’okunywa ebitamiiza nga ogeza kuby’okunywa ebyabulijjo gamba nga bbiya, wayini, vodika, n’ebiri nga ebyo. Ebiddibwamu biwandiike mu ngeri emanyiddwa wano, ebibuuzo ebituufu biteeke mu ka bokisi akali kuddyo.

Read questions as written. Record answers carefully. Begin the AUDIT by saying “Now I am going to ask you some questions about your use of alcoholic beverages during this past year.” Explain what is meant by “alcoholic beverages” by using local examples of beer, wine, vodka, etc. Code answers in terms of “standard drinks”. Place the correct answer number in the box at the right.

| **No.** | Question | Possible scores | Score | Answer code |
| --- | --- | --- | --- | --- |
| **1.** | Emirundi emeka gy’otera okukozesa eky’okunywa ekitamiiza?  How often do you have a drink containing alcohol? | 0 = never  1 = monthly or less  2 = 2 to 4 times/month  3 = 2 to 3 times/week  4 = 4 or more times/week | |**__**|  If score 0, go to blood sample | AUDRINK |
| **2.** | Onywa ebipimo bimeka eby’omwenge buli lwoba onywedde? Okugeza ecupa oba egilasi meka?  How many drinks containing alcohol do you have on a typical day when you are drinking? | 0 = 1 or 2  1 = 3 or 4  2 = 5 or 6  3 = 7, 8, or 9  4 = 10 or more | |**__**| | AUNUM |
| **3.** | Emirundi emeka gy’onywa ebipimo ebiwera omukaaga oba okusingawo mulunywa olumu?  How often do you have six or more drinks on one occasion? | 0 = never  1 = less than monthly  2 = monthly  3 = weekly  4 = daily or almost daily | |**__**|  If combined score for questions 2 and 3 = 0, go to questions  9 and 10 | AUSIX |
| **4.** | Emirundi emeka omwaka oguyise bweweesanga olemererwa okulekeraawo okunywa bwobanga otandise okunywa?  How often during the last year have you found that you were not able to stop drinking once you had started? | 0 = never  1 = less than monthly  2 = monthly  3 = weekly  4 = daily or almost daily | |**__**| | AUSTOP |
| **5.** | Emirundi emeka omwaka oguyise bwewesanga nga olemereddwa okutuukiriza obuvunaanyizibwa obwabulijjo olw’okunywa?  How often during the last year have you failed to do what was normally expected from you because of drinking? | 0 = never  1 = less than monthly  2 = monthly  3 = weekly  4 = daily or almost daily | |**__**| | AUFAIL |
| **6.** | Emirundi emeka omwaka oguyise gy’eweessanga nti osooka kunywako mwenge kumakya nga tonnabako ky’okola oluvannyuma lwo kunywa ennyo eggulo limu?  How often during the last year have you needed a first drink in the morning to get yourself going after a heavy drinking session? | 0 = never  1 = less than monthly  2 = monthly  3 = weekly  4 = daily or almost daily | |**__**| | AUMORN |
| **7.** | Emirundi emeka omwaka oguyise gye weewulira nga azzizza omusango oba okwejjusa mu mitima oluvannyuma lw’okunywa ennyo?  How often during the last year have you had a feeling of guilt or remorse after drinking? | 0 = never  1 = less than monthly  2 = monthly  3 = weekly  4 = daily or almost daily | |**__**| | AUGUILT |
| **8.** | Emirundi emeka omwaka oguyise bweweesanga nga tosobola kujukira ebyabaddewo ekiro ekyayise kubbanga wabadde onywedde?  How often during the last year have you been unable to remember what happened the night before because you had been drinking? | 0 = never  1 = less than monthly  2 = monthly  3 = weekly  4 = daily or almost daily | |**__**| | AUMEM |
| **9.** | Ggwe oba omuntu omulala mwali mutusiddwako obuvunne olwokuba nti ggwe wali onywedde?  Have you or someone else been injured as a result of your drinking? | 0 = no  2 = yes, but not in the last year  4 = yes, during the last year | |**__**| | AUINJUR |
| **10.** | Waliwo omuntu yenna okugeza ow’oluganda, mukwano gwo, oba omusawo eyali afudde yo kunnywayo oba eyali agambye nti okendeezeeko kunnywayo?  Has a relative or friend or a doctor or another health worker been concerned about your drinking or suggested you cut down? | 0 = no  2 = yes, but not in the last year  4 = yes, during the last year | |**__**| | AUCONC |
|  | **Record total score**  (sum of scores for individual items) | If total is 8 or more, offer the participant counselling and referral. | |__|__| | AUSCORE |

**PEAK EXPIRATORY FLOW RATE** |__|__|PEFR

**BLOOD SAMPLE**

5ml with vacutainer for HIV test

1 = specimen obtained, 2 = specimen to be obtained later, 7 = refused, 9 = failed |__| VAC

Interviewer code of the person taking the blood sample if different from the interviewer |__|__|DINTCODE

**|__|__|__|__|__|__|__|__|** LABNO

52. Wandyagadde okumanya ebiva mukukebera omusaayi gwo? |__|KVCT

Would you like to know the result of this HIV test?

1 = yes, 2 = no, 8 = don’t know/not sure

**TREATMENT**

Instruction to interviewer: please record here if any treatment provided to participant on the spot

Diagnosis:

Treatment:
